# Understanding 2-(Nitromethylene)hexahydropyrimidin-5-ol Reaction Processes and NMR Spectroscopy: A Theoretical and Experimental Investigation

**DOI:** 10.1021/acsomega.4c08242

**Published:** 2024-12-21

**Authors:** Ramon S. da Silva, Diego P. Sangi, Rodrigo G. Amorim

**Affiliations:** † Departamento de Física - Instituto de Ciências Exatas - ICEx, Universidade Federal Fluminense, Volta Redonda, Rio de janeiro 27213-145,Brazil; ‡ Departamento de Química - Instituto de Ciências Exatas - ICEx, Universidade Federal Fluminense, Volta Redonda, Rio de janeiro 27213-145,Brazil

## Abstract

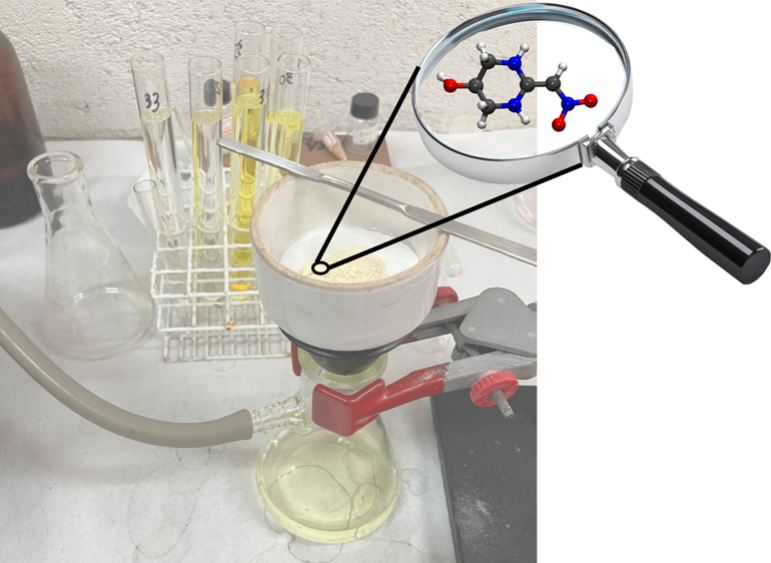

Ketene dithioacetals have significant applications in various fields, including natural products, pharmaceuticals, agrochemicals, and corrosion inhibitors. These compounds are highly valued for their reactivity and ability to participate in a wide range of organic syntheses. In this context, the reaction between 1,3-diaminopropan-2-ol and 1,1-bismethylsulfanyl-2-nitroethylene has been studied experimentally and theoretically by using density functional theory (DFT) calculations. A theoretical mechanism of formation of two possible products, 2-(nitromethylene)hexahydropyrimidin-5-ol (with a six-membered heterocycle) and (2-(nitromethylene)oxazolidin-5-yl)methanamine (with a five-membered heterocycle), is for the first time predicted. The present DFT results indicate that both mechanisms are exothermic, with energy barriers approximately 20 kcal/mol higher than those of the reactants. Among the two, the formation of 2-(nitromethylene)hexahydropyrimidin-5-ol is energetically more favorable. This compound was synthesized and analyzed by different experimental techniques (IR, nuclear magnetic resonance (NMR), and high-resolution mass spectrometry). The ^1^H and ^13^C NMR chemical shifts of 2-(nitromethylene)hexahydropyrimidin-5-ol were calculated using the GIAO/B3LYP, showing good agreement with our experimental observations. These findings highlight an important match between experimental results and theoretical predictions, offering deeper insights into ketene dithioacetal reactions. The new data and contributions are expected to generate significant interest in future applications.

## Introduction

**Figure 1:**
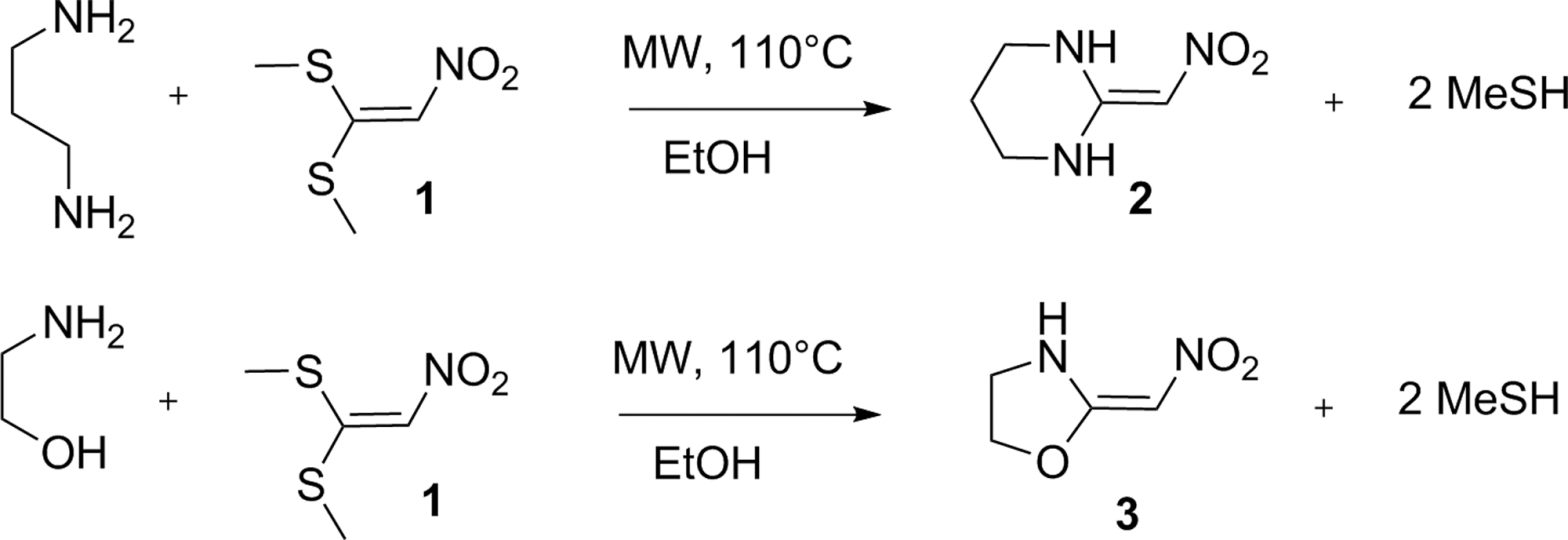
Synthesis of 2-nitromethylenehexahydropyrimidine (**2**) and 2-nitromethyleneoxazolidine (**3**).

Ketene dithioacetals are important building blocks in organic synthesis, due to their ability to be substrates of many reactions,^1,2^ including to produce heterocyclic scaffolds with different properties and applications, comprising new prototypes of pharmaceuticals,^3,4^ agrochemicals,^5^ and potential corrosion inhibitors.^6,7^ Particularly, polarized ketene dithioacetals can be used as electrophiles to double vinylic substitutions with diamines and aminols in a simple way to obtain 1,3-diazo and 1,3-oxazo heterocycles of the imidazolidines, oxazolidines, hexahydropyrimidines, oxazinanes, and benzoxazole classes.^5,8^ In recent years, we have applied ketene dithioacetals in syntheses of compound heterocycles with diverse biological activities and applications. Thus, it was found that 1,1-bismethylsulfanyl-2-nitroethylene (**1**) allows the construction of five- and six-membered rings, as shown in the synthesis of 2-nitromethylene hexahydropyrimidine (**2**) and 2-nitromethylene oxazolidine (**3**) (Figure 1).^6−8^

**Figure 2:**
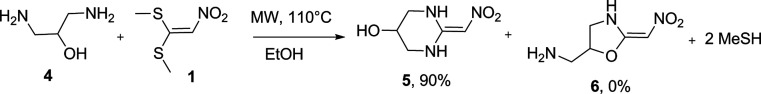
Double vinylic substitution of 1,1-bis-methylsulfanyl-2-nitroethylene (**1**) with 1,3-diaminepropan-2-ol.

Owing to our interest in the synthesis and physicochemical properties of these heterocyclic materials, we decided to investigate how the annelation reaction works, employing 1,3-diaminepropan-2-ol (**4**) as a nucleophile, which could enable the formation of the hexahydropyrimidine (**5**) and/or oxazolidinic (**6**) derivative. To our surprise, we observed only the formation of compound **5**, as shown in Figure 2. The experimental results provided by our setup suggest a preference or thermodynamic control favoring the formation of compound **5** over compound **6** under the reaction conditions employed. Theoretical investigations into the reaction mechanism may provide insights into the underlying factors driving this selectivity. They could expand the scope of heterocyclic ring formation using similar nucleophilic annelation strategies, such as related catalyzed synthesis of pyridines.^9−11^

In this work, we provide a theoretical view of molecular properties and formation mechanisms involved in the reactions shown in Figure 2 by employing density functional theory (DFT) calculations. From the experimental side, nuclear magnetic resonance (NMR) spectroscopy is utilized to characterize the molecular structure of 2-(nitromethylene)hexahydropyrimidin-5-ol (**5**), since this technique allows us to determine the arrangement of atoms within molecules by analyzing the magnetic properties of atomic nuclei. In this sense, DFT calculations are used to compute the nuclear magnetic shielding tensors for hydrogen (^1^H) and carbon (^13^C) nuclei in the molecule. The shielding tensor provides information about the local magnetic environment of the nuclei and can be compared with the experimental NMR shielding tensors. To evaluate the influence of functional and basis sets on the DFT results, the benchmark using different combinations was also tested. Time-dependent density functional theory (TDDFT) is used to calculate the energy gap between the ground singlet state and excited states (singlet and triplet) of the compounds. TDDFT is an extension of DFT that allows for the investigation of excited states and electronic transitions. One can estimate optical characteristics and molecular photochemical reactions by looking at the energy gaps between the various electronic states.

## Synthetic Methodology to Produce 2-(Nitromethylene)hexahydropyrimidin-5-ol (**5**)

Reaction assisted by microwave irradiation was conducted using an Anton Paar Monowave 300 device. Thin-layer chromatography analyses were performed using commercial aluminum plates coated with a 0.2 mm layer of silica gel from the Macherey-Nagel brand and developed under ultraviolet light (254 nm). The melting point was measured using a Gehaka PF1500 Farma instrument. Infrared spectra were obtained using a Bruker FT-IR Vertex 70 spectrophotometer in attenuated total reflectance (ATR) mode, covering the range from 400 to 4000 cm^–1^. NMR analyses were conducted by using a Varian VNMRS 500 MHz spectrometer. Mass spectroscopy was performed using a Shimadzu GCMS-QP2010 Plus and a Waters/Micromass UPLC-QTof-MS instrument for high-resolution analysis.

1,1-Bis-methylsulfanyl-2-nitroethylene (165 mg, 1.0 mmol), 1,3-diaminepropan-2-ol (90 mg, 1.0 mmol), and ethanol (3 mL) were placed in a glass tube, sealed, and irradiated during 60 min in an Anton Paar Monowave 200 microwave reactor at 110 °C. After cooling, the 2-(nitromethylene)hexahydropyrimidin-5-ol precipitated as a yellow solid that was isolated by filtration and purified by washing with cold ethanol.

yield 90%. ^1^H NMR (DMSO-d_6_): δ 8.90–8.74 (m, 2H), 6.28 (s, 1H), 5.31 (d, *J* 2.5 Hz, 1H), 4.06–3.98 (m, 1H), 3.39–3.32 (m, 2H), 3.20–3.10 (m, 2H). ^13^C NMR (DMSO-d6): δ 154.23, 98.47, 58.38, 44.57. IR (cm^–1^): 3220, 3134, 1603, 1498, 1403, 1314, 1147, 1102, 1110, 723, 670, 576, 426. HRMS (ESI): Calcd for C_5_H_10_N_3_O_3_ [M + H]^+^ = 160.0723, found 160.0776.

## Computational Details

DFT calculations were carried out using the ORCA code.^12^ Initially, we employed Becke3-Lee–Yang–Parr (B3LYP) for the geometry optimizations and reaction energies. To ensure accurate results, the very tight convergence criterion (very tight square root of failure) was adopted during the calculations. All compounds studied here were optimized separately using three classes of basis sets. These basis sets include Ahlrichs’ def2-TZVP basis set,^13^ Pople’s split-valence triple-ζ basis set 6-311G(d,p), and the cc-pVTZ basis set of Dunning. Stationary points were confirmed to be minima (on a potential energy hypersurface) through frequency calculations, which showed the absence of imaginary frequencies in some cases.

The excitation energies of the first excited singlet state (S_1_) and first triplet excited state (T_1_) were calculated based on the TDDFT method as implemented in the ORCA code. Analysis of the photophysical properties between singlet–singlet S_0_ → S_1_ and singlet–triplet S_0_ → T_1_ transitions was obtained employing the B3LYP functional.

NMR chemical shifts were calculated using the gauge-independent atomic orbital (GIAO) method.^14^ All ^13^C and ^1^H NMR chemical shifts were computed with reference to tetramethylsilane (TMS), obtained at the same level of theory, according to the following expression:
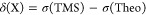
1The solvent effect of dimethyl sulfoxide (DMSO) used in the experiment was included in the theoretical calculations using the conductor-like polarizable continuum model (CPCM).^15^ For comparison purposes, calculations were also performed using another functional (BP86) and basis set (pcSseg-2^16^).

## Results and Discussion

### Synthesis of 2-(Nitromethylene)hexahydropyrimidin-5-ol (**5**)

**Figure 3:**
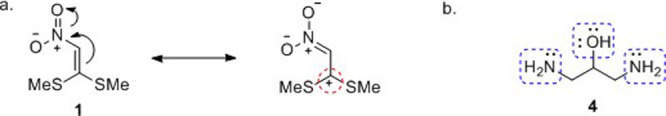
(a) Electrophile center of 1,1-bis(methylsulfanyl)-2-nitroethylene (**1**). (b) Nucleophile centers of 1,3-diaminepropan-2-ol (**4**).

**Figure 4:**
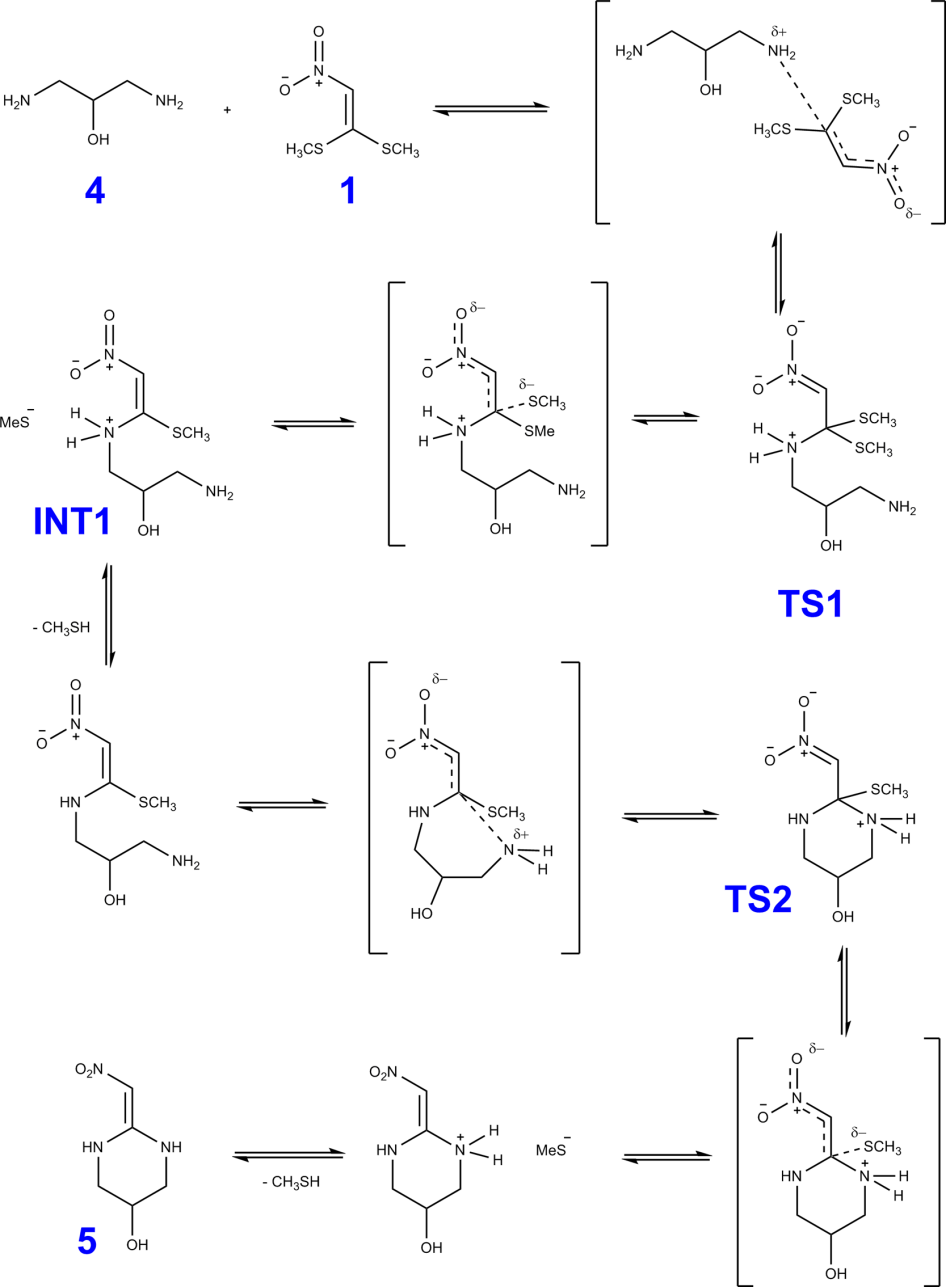
Mechanism to synthesis of 2-(nitromethylene)hexahydropyrimidin-5-ol (**5**).

Due to the presence of the electron-withdrawing group nitro in 1,1-bis(methylsulfanyl)-2-nitroethylene (**1**), this compound is an electrophile used to produce heterocyclic compounds of five and six members through double vinylic substitution of methylsulfanyl groups (see Figure 3). 1,3-Diaminepropan-2-ol (**4**) has three nucleophilic centers (Figure 3), where the amine groups are separated by three carbons and amines are separated from the hydroxyl group by two carbons, so it could be expected that the reaction between compounds **1** and **4** could produce 2-(nitromethylene)hexahydropyrimidin-5-ol (**5**) and (2-(nitromethylene)oxazolidin-5-yl)methanamine (**6**) (Figure 2). Due to the higher nucleophilicity of amino groups than hydroxyl, in the first step of the reaction, one of the amino groups does the addiction on the carbon electrophilic β nitro to in sequence eliminate methanethiol, finishing the mono vinylic substitution and producing the transition state, **TS1**, as shown in Figure 4. On the second part of the reaction, the second vinylic substitution occurs by the addition of another amino or the hydroxyl group with the second elimination of methanethiol.

**Figure 5:**
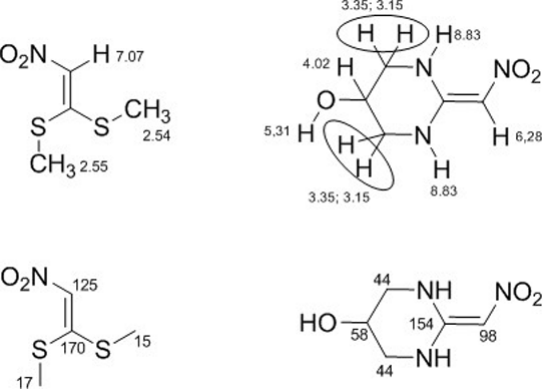
Top: ^1^H chemical shifts (in ppm) of compounds **1** and **5**. Bottom: ^13^C chemical shifts of compounds **1** and **5**.

After the experimental procedure to synthesize heterocycles of five and six members, we verified by chromatography that only one compound was produced. The NMR spectra results for 1,1-bis-methylsulfanyl-2-nitroethylene and 2-(nitromethylene)hexahydropyrimidin-5-ol are presented in Figures S1 and S2 of the Supporting Information (SM), respectively. ^1^H NMR experiments (in SM) show that two singlets in 2.54 and 2.53 ppm referents to methylsulfanyl groups were substituted to a multiplet integrated to 1H in 4.06–3.98 ppm, which is a typical chemical shift for carbinolic hydrogens, and two multiplets integrated to 2H each one in 3.39–3.32 and 3.20–3.10 ppm, which is the typical shift to alfa amino hydrogens (there are two signals here due to relative position to hydroxy group). Additionally, we verified the presence of a multiplet integrated to 2H in 8.90–8.74 ppm referents to hydrogens of the two H–N groups that are highly deshielded because delocalizations of the nitrogens electrons with sp^2^ carbons, a duplet in 5.31 ppm to hydrogen of the hydroxyl group, and a singlet in 6.28 ppm integrated to ^1^H referent to olefinic and α-nitro hydrogen that was also present in compound **1** (see Figure 5).

^13^C NMR experiments (in SM) show that the signals referents to olefinic carbons in 154 and 98 ppm are shielded in compound **5** in comparison with compound **1** (170 and 125 ppm), as expected by this kind of transformation. On the other side, the signals in 17 and 15 ppm referents to carbons of methylsulfanyl groups (compound **1**) were substituted by signals in 58 and 44 ppm, which are typical shifts to carbons bonded to oxygen and nitrogen, respectively (Figure 5). Interestingly, there is only one signal (44 ppm) referent to CH_2_NH; it seems to indicate that the alkene carbon has a very low barrier for rotation due to the resonance with nitro and two N–H groups.

These data of NMR experiments are very consistent to confirm that compound **5** was produced instead of compound **6**. Since compound **6** should show two different signals to hydrogens of N–H and NH_2_ groups on ^1^H NMR and two different signals to carbons of CH_2_–NH and CH_2_–NH_2_ groups on ^13^C NMR (in concord to theoretical NMR to compound **6**). The identification of compound **5** was further confirmed through high-resolution mass spectrometry (HRMS) and infrared (IR) spectroscopy, as detailed in parts S3 and S4 of the SM.

### Theoretical Results on Structural and Molecular Properties

**Figure 6:**
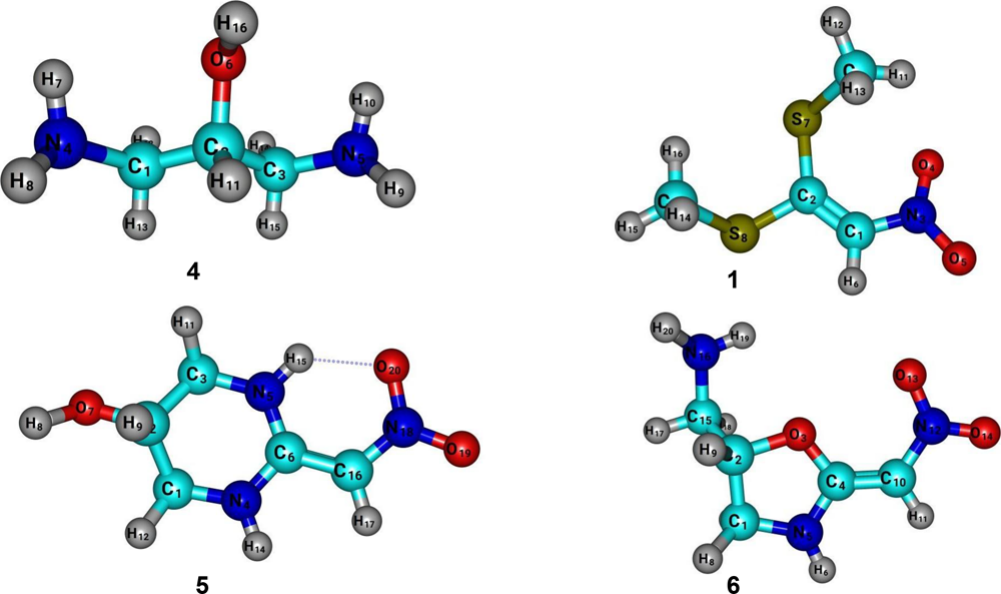
DFT optimized structures of compounds **4** [1,3-diaminopropan-2-ol], **1** [1,1-bismethylsulfanyl-2-nitroethylene], **5** [2-(nitromethylene)hexahydropyrimidin-5-ol], and **6** [(2-(nitromethylene)oxazolidin-5- yl)methanamine].

The relevant bond lengths and angles for nonplanar structures were computed using the DFT-B3LYP functional with different basis sets. The obtained results are presented in Tables S1–S9 of the Supporting Information. For convenience, optimized geometries with atom labeling at the B3LYP/def2-TZVP level of theory are presented in Figure 6. First, we considered the structure of 1,3-diaminopropan-2-ol (compound **4** in Figure 6). The calculated C_1_–C_2_ and C_2_–C_3_ bond distances at the B3LYP/6-311G(d,p) (B3LYP/def2-TZVP) are 1.535 Å (1.532 Å) while the C_1_–C_2_–C_3_ bond angle is 113.6° (112.2°). The results obtained using the 6-311G(d,p) and def2-TZVP basis sets differ by only 0.003 Å. The C_1_–N_4_ and C_3_–N_5_ distances are estimated to be 1.462 (1.459 Å). All N–H bond distances in this species are ≈1.011 Å. The B3LYP/6-311G(d,p) calculations reveal that the C_2_–O_6_ bond length of 1.478 Å is larger than the experimental data of 1.1283 Å^17^ obtained for free carbon monoxide. Additionally, the bond angle formed between C_2_–O_6_–H_16_ is 111.0°, which differs by about 1.5° from the def2-TZVP and VTZ basis sets. As can be seen in Table S1 of the SM, the dipole moment, μ, increases from 2.788 D (VTZ) to 3.312 D (6-311G(d,p)). The total energies obtained by the DFT calculations were −304.848133 for B3LYP/6-311G(d,p) and −304.989487 for B3LYP/VTZ.

The atomic charge values were calculated using Mulliken population analysis, as implemented in the ORCA code, to determine the charge distribution within these molecules. At the B3LYP/VTZ [B3LYP/def2-TZVP] level of theory, the Mulliken atomic charges for the nitrogen atoms in 1,3-diaminopropan-2-ol are −0.33 [−0.38e], while for the oxygen atom, it is −0.37 [−0.40e]. These results are consistent with our experimental observations, indicating that these sites act as nucleophilic centers. On the other hand, the O-bound carbon atom has an atomic charge of +0.08e. The remaining ten hydrogen atoms have positive charges. The most positive charge among them was calculated for the H-bound oxygen atom, which is +0.20 [+0.27e]. The atomic charges for the hydrogen atoms in the NH_2_ group are close to +0.12 [+0.18e].

The longest bond lengths for the structure of 1,1-bismethylsulfanyl-2-nitroethylene (compound **1** in Figure 6) are observed for the C_2_–S_7_ and C_2_–S_8_ bonds, with values of 1.75 and 1.78 Å, respectively. Conversely, the shortest bond lengths are found for the C–H bonds, which have values around 1.09 Å. The average N_3_–C_1_–C_2_ bond angle, calculated from our DFT calculations, is 127.2°. The NO_2_ group in this compound exhibits an average O_5_–N_3_–O_4_ bond angle of 124.0° and an N=O bond distance of approximately 1.23 Å. These results deviate by 10.0° and 0.4 Å, respectively, from the experimental and theoretical data reported for the ground electronic state of the free radical.^18^ We compute a dipole moment of 5.594 D using the def2-TZVP basis set, which is comparable to the dipole moment reported for 2-(4-methox-ybenzoyl)-3,3-bis(methylsulfanyl)prop-2-enal (5.65 D).^19^ With the current knowledge, we also determine the relationship that follows μ(**1**) ≈ 2 μ(**4**).

Based on Mulliken population analysis, we find ten positive charge atoms in the charge distribution of 1,1-bismethylsulfanyl-2-nitroethylene. The Mulliken atomic charges for the oxygen atoms in the nitro group are approximately −0.31e, while for the nitrogen atom, it is +0.36e at the B3LYP/VTZ level of theory. The two methanethiolates are connected to a carbon atom with a positive charge (approximately +0.10e). The hydrogen atoms in the methanethiolates all have positive charges, with an average charge of +0.12e.

We verify both experimentally and theoretically that the chemical reaction between 1,3-diaminopropan-2-ol and 1,1-bismethylsulfanyl-2-nitroethylene can produce a cyclic compound, 2-(nitromethylene)hexahydropyrimidin-5-ol (six-membered ring) or (2-(nitromethylene)oxazolidin-5-yl)methanamine) (five-membered ring), together with two molecules of methanethiol through a transition state (Figure 4). In our case, we only detected experimentally the product containing 2-(nitromethylene)hexahydropyrimidin-5-ol plus two methanethiols. Additionally, it could possess sufficient energy to form (2-(nitromethylene)oxazolidin-5-yl)methanamine. However, upon analyzing Tables S4 and S5 of the Supporting Information, we note a large energy difference of approximately 12.7 kcal/mol (B3LYP/VTZ) [13.6 kcal/mol; B3LYP/def2-TZVP] between them. Strictly speaking, this result suggests a significant Boltzmann population favoring the formation of 2-(nitromethylene)hexahydropyrimidin-5-ol over the (2-(nitromethylene)oxazolidin-5-yl)methanamine.

The electronic state of this structure is a closed-shell singlet state. The lengths of its C–C and C–N bonds are around 1.234 and 1.367 Å, respectively. We computed the N=O bond distance in the nitro group in the range of 1.226 (VTZ) to 1.235 (6-311G(d,p)) Å, depending on the basis set used. The N–H bond distances in the amino group, such as N_17_–H_32_ and N_21_–H_24_, were calculated at the def2-TZVP basis set to be 1.018 and 1.014 Å, respectively. The longest bond distances computed for this structure are attributed to the C_2_–S_7_ and C_2_–S_8_ bonds (∼1.874 Å, VTZ). This value is approximately 0.1 Å larger than that for 1,1-bismethylsulfanyl-2-nitroethylene, indicating that the formation of two molecules of methanethiolate (CH_3_S) is favorable from this structure.

Notably, this compound is a promising candidate for proton transfer reactions due to the presence of an N–H···O hydrogen bond, consistent with the IUPAC definition.^20^ Studies on hydrogen bonding are attracting considerable interest in fields such as biological processes^21^ and materials science.^22^ We calculated the following geometrical parameters: an N_17_–H_31_···O_4_ bond angle of 145.5°, an H_31_···O_4_ bond distance of 1.512 Å, and a separation distance N_17_–O_4_ of 2.493 Å, which are in line with the criteria recommended by the IUPAC. After several tests, the minimum N–O separation in the N–H···O configuration was estimated by Nikolaienko et al.^23^ to be ≈2.82 Å, in qualitative agreement with our value.

The atomic charge at the C_1_ atom is highly negative, with a value of −0.36e (def2-TZVP basis set). The Mulliken atomic charges of the hydrogen atoms (H_11_, H_12_, and H_13_) in the vicinity of C_9_ were calculated to be approximately +0.14e. N_17_ and N_21_ have negative charges of −0.24e and −0.42e, respectively, except for the N_3_ atom, which has a positive charge of +0.23e due to the presence of oxygen atoms O_4_ (−0.40e) and O_5_ (−0.28e).

In the case of 2-(nitromethylene)hexahydropyrimidin-5-ol (compound **5** in Figure 6), the ring (six-membered) is essentially nonplanar with a dihedral angle of O_7_–C_2_–C_1_–N_4_ equal to 173.1° at the VTZ basis set. The distortion occurs due to the interaction of the hydroxyl group (−OH) with the ring, resulting from the high electronegativity of the oxygen atom. This interaction can change the electronic distribution within the ring due to its electron-donating nature. However, the ring structure itself is not destroyed. The nitro group of this molecule is planar, with a bond angle of ≈122.0° for the bond angle of O_20_–N_18_–O_19_ of ≈122.0°. The C–H bond distances were calculated to be approximately 1.077 Å in general. The obtained N–O bond distance is estimated to be 1.233 Å, which is 0.08 Å larger than that of the free NO radical (1.153 Å).^24^

The Mulliken atomic charges for the nitrogen atoms N_4_ and N_5_ are −0.46e (−0.28) at the VTZ (def2-TZVP) basis set, while the N_18_ atom has a positive charge of +0.22 (+0.23e). Mulliken charges of every H atom are all positive. For example, H_8_ to H_15_ atoms of compound **5** are computed to be 0.24, 0.12, 0.13, 0.13, 0.11, 0.13, 0.23, and 0.26e, respectively. The Mulliken charges of oxygen atoms are −0.39e. The dipole moment is 8.752 D, and the optimization energy is −585.619566 hartree at the cc-pVTZ Dunning’s correlation consistent basis set. The same quantities were also calculated at the def2-TZVP basis set. We compute a dipole moment of 9.116 D and a total energy of −585.671971 hartree. Additionally, we calculated the IR spectrum of 2-(nitromethylene)hexahydropyrimidin-5-ol. A comparison with the experimental data is shown in Figure S4 of the Supporting Information.

The optimized structure of (2-(nitromethylene)oxazolidin-5-yl)methanamine) computed using the B3LYP/VTZ level of theory is shown in Figure 6 [compound **6**]. The C_4_=C_10_ bond distance [1.359 Å] represents a purely double bond, forming an angle of 124.9° with the N_12_ atom belonging to the nitro group. We calculated the N–O bond distance in this group to be approximately 1.234 Å. The C_1_–C_2_ bond distance in the ring is ∼1.530 Å at VTZ and def2-TZVP basis sets. The C_4_–N_5_ and C_1_–N_5_ bond distances are 1.372 and 1.459 Å, respectively, suggesting a torsion in the C_1_ atom due to hydrogens H_7_ and H_8_. In this sense, we compute the dihedral angle between C_4_–N_5_–C_1_–H_7_ of 93.7°. As a comparison, the dihedral angle formed between the atoms C_4_–C_10_–N_12_–O_13_ is 0.5°, showing a quasi-planar structure.

The dipole moment of compound **6** is similar to that of compound **5**. We computed a value of 8.592 D at the B3LYP/VTZ level of theory, consistent with the dipole moment calculated for compound **5**. This suggests that the Mulliken charges of this compound may be analogous to those of 2-(nitromethylene)hexahydropyrimidin-5-ol. The Mulliken charges of every hydrogen atom belonging to compound **6** are all positive, ∼ +0.12e. The oxygen atom, O_3_, in the ring, has noted a negative charge of −0.21e, while the carbon atoms C_1_, C_2_, and C_4_ have charges of −0.11e, +0.09e, and +0.31e, respectively. In the nitro group, the Mulliken charge is +0.35e for N_12_ and approximately −0.31e for O_13_ and O_14_. In the amine group, the Mulliken charge is −0.32e for N_16_ and approximately +0.15e for hydrogen atoms H_19_ and H_20_.

At last, Garrison and Sandler^25^ studied the molecular structure of methanethiol and reported bond distances of R(S–H) = 1.34 Å, R(C–S) = 1.819 Å, and R(C–H) = 1.09 Å. They also calculated H–S–C and H–C–H bond angles of 96.5 and 109.8°, respectively. These quantities compare well with the present B3LYP results.

### Frontier Molecular Orbitals and Chemical Reactivity

**Figure 7:**
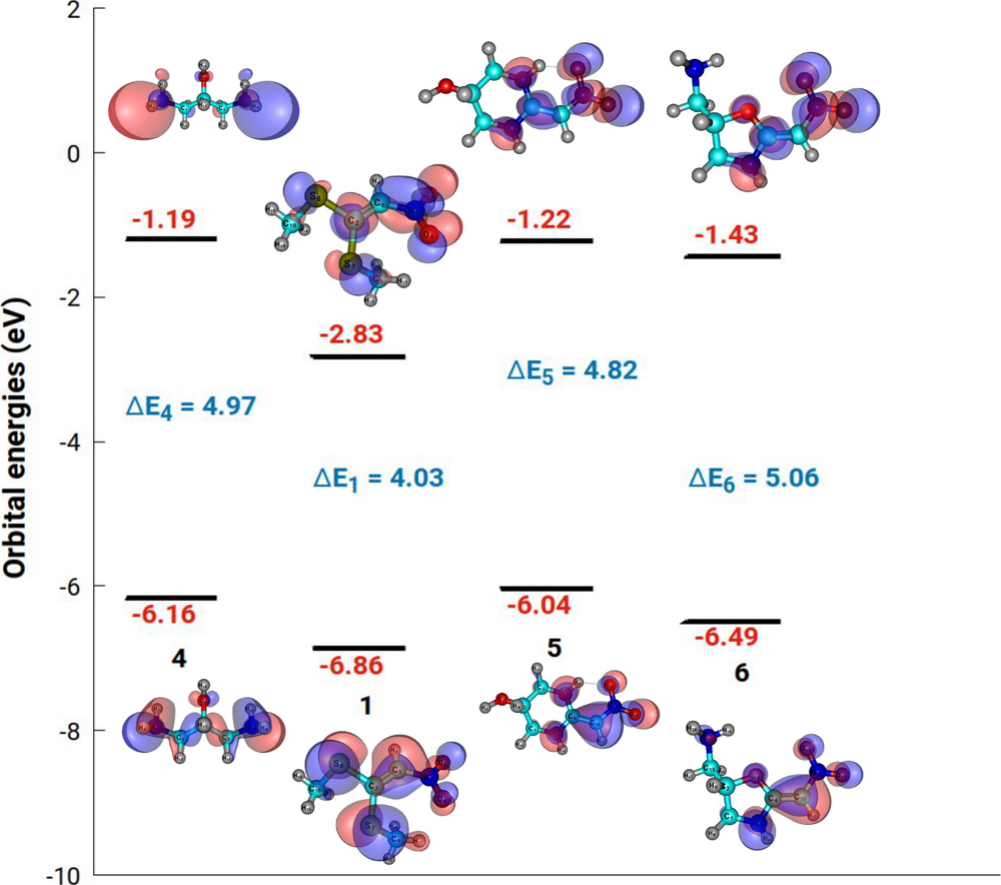
Calculated energy level diagram of HOMOs and LUMOs at DFT-B3LYP/VTZ for the molecules involved in the chemical reaction. The label and molecule names are the same as those presented in Figure 6.

The highest occupied molecular orbital (HOMO) and lowest unoccupied molecular orbital (LUMO) are depicted in Figure 7 together with their respective energy gap values. A molecule with a high energy gap between HOMO and LUMO exhibits high molecular stability and low chemical reactivity. This stability arises because a large energy gap implies a significant energy barrier for electronic transitions, thereby influencing the molecule’s reactivity in chemical reactions. Conversely, a small energy gap suggests a molecule with a high chemical reactivity.

The DFT reactivity descriptors such as electronegativity (χ), chemical hardness (η), chemical potential (μ_0_), softness (*S*), and electrophilicity index (ω) were calculated with the help of the energy gap between HOMO–LUMO for these structures according to equations^26^^,^^27^:
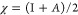
2
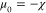
3
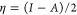
4
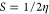
5
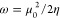
6

The ionization potential (*I*) is the energy required to remove an electron from a neutral molecule to form a positively charged ion. This process typically involves removing an electron from the HOMO of the molecule. Therefore, the energy of the HOMO is directly related to the ionization potential according to *I* = −*E*_HOMO_. Similarly, electron affinity (*A*) is the energy released when an electron is added to a neutral molecule to form a negatively charged ion. The energy of the LUMO is directly related to the electron affinity according to *A* = −*E*_LUMO_. All of the calculated values of DFT reactivity descriptors are shown in Table 1.

**Table 1:** Quantum Chemical Parameters, in eV, Calculated at the B3LYP/VTZ Level of Theory^a^

parameter	4	1	5	6
*I*	6.16	6.86	6.04	6.49
*A*	1.19	2.83	1.22	1.43
χ	3.67	4.85	3.63	3.96
η	2.49	2.02	2.41	2.53
μ_0_	–3.67	–4.85	–3.63	–3.96
ω	2.70	5.82	2.73	3.09
*S*	0.20	0.25	0.20	0.19

a As seen in Figure 6, the label and molecular names are the same.

All DFT calculations were performed using the B3LYP/VTZ level of theory. The frontier orbital energy gaps for compounds **4**, **1**, **5**, and **6** were found to be 4.97, 4.03, 4.82, and 5.06 eV, respectively. We can conclude that the stability of the studied compounds follows the trend of **6** > **4** > **5** > **1**. The compound **1** has the lowest energetic gap, while the compound **6** has the highest energetic gap. This trend indicates that the (2-(nitromethylene)oxazolidin-5-yl)methanamine is slightly more stable (less reactive) than 2-(nitromethylene)hexahydropyrimidin-5-ol and tends to have a longer lifetime. Note that the compound **1** is relatively softer (*S* = 0.21 eV) than other compounds. The hardness of the studied compounds follows the steady trend of the energy gap.

A high value of chemical potential and electrophilicity index is indicative of a good electrophile. As seen in Table 1, the present DFT results quantitatively confirm the 1,1-bis(methylsulfanyl)-2-nitroethylene, compound **1**, as the most electrophilic species among the compounds analyzed, with μ_0_ = −4.85 eV and ω = 5.82 eV. For compound **4**, the calculated value for the global electrophilicity index was 2.70 eV, approximately half of ω for **1**, indicating that **4** is more nucleophilic than **1** in line with our experimental results. On the other hand, the large band gap of 5.06 eV makes the (2-(nitromethylene)oxazolidin-5- yl)methanamine more electrophilic than the 2-(nitromethylene)hexahydropyrimidin-5-ol, with μ_0_ = −3.96 eV and ω = 3.09 eV.

### Overview on Photophysical Properties

The photophysical properties calculated in this work using the TDDFT methodology are listed in Table 2. All species analyzed here present a closed-shell singlet ground state (S_0_) and a low-lying triplet (T_1_) excited state, resulting in a positive energy separation, denoted as the Δ*E*(S_0_–T_1_). Singlet–triplet transitions are spin-forbidden by selection rules; however, they can occur due to spin–orbit coupling.^28^ In general, the first excited triplet state (T_1_) is lower in energy than the first excited singlet state (S_1_), and it cannot absorb light. Considering a Jablonski diagram in molecular spectroscopy, one way to populate the T_1_ state is through UV light absorption via the S_0_–S_1_ transition, followed by a nonradiative transition to the triplet state.^29^ Once the T_1_ state is populated, it can be responsible for the phosphorescence phenomenon at low temperatures.^29^ A literature survey reveals that quantitative studies of the intensity of the S_0_–S_1_ transition in these species are very scarce or even nonexistent, despite such data being crucial for understanding the spectroscopy of these species.

**Table 2:** Benchmarking of Excitation Energy, in eV, for the Compounds Analyzed^a^

	4		1		5		6	
method	Δ*E*(S_0_–S_1_)	Δ*E*(S_0_–T_1_)	Δ*E*(S_0_–S_1_)	Δ*E*(S_0_–T_1_)	Δ*E*(S_0_–S_1_)	Δ*E*(S_0_–T_1_)	Δ*E*(S_0_–S_1_)	Δ*E*(S_0_–T_1_)
B3LYP/def2-TZVP	5.438	5.261	3.568	2.530	4.066	2.933	2.083	1.599
	7.01(−3)		2.11(−2)		2.23(−5)		1.12(−6)	
B3LYP/6-311G(d,p)	5.573	5.373	3.588	2.558	4.110	2.986	2.058	1.560
	8.95(−3)		8.25(−3)		1.17(−4)		5.47(−7)	
B3LYP/VTZ	5.537	5.351	3.640	2.676	4.128	2.981	2.069	1.583
	6.96(−3)		1.18(−2)		1.06(−4)		3.42(−7)	

a Oscillator strength values are given in the second line of each base set, and the number in parentheses indicates the power of ten by which the preceding number is to be multiplied.

In terms of molecular orbitals, singlet–singlet and singlet–triplet transitions can be represented by the schemes HOMO → LUMO and HOMO → LUMO + 1, respectively. As can be seen in Table 2, the choice of basis set can significantly influence the values of photophysical properties. For example, structure **4** exhibits a vertical singlet–singlet excitation within the energy range of 5.44–5.57 eV (≈222 nm). This transition has an *n*–π* characteristic due to the charge-transfer nature of such a transition, presenting a large contribution from the amino group. The corresponding oscillator strength, *f*, for this transition is 6.96 × 10^–3^ at the B3LYP/VTZ and 7.01 × 10^–3^ at B3LYP/def2-TZVP. The present calculations reveal that the vertical S_0_–T_1_ energy difference is within the range of 5.26 to 5.35 eV.

The S_1_(*n*–π*) state in 1,1-bismethylsulfanyl-2-nitroethylene is located at 3.57 eV (λ = 347 nm) with the def2-TZVP basis set, but at 3.64 eV (λ = 340 nm) with the Dunning basis set cc-pVTZ. According to our computations, this transition is the most intense among the species analyzed with *f* ≈ 10^–2^ with a relatively large portion of charge transfer from the nitro group (−NO_2_). From a spectroscopic point of view, species **1** is expected to exhibit strong UV light absorption. The singlet–triplet excitation energy increases from 2.53 to 2.68 eV, which indicates a significant sensitivity on the basis set employed. Additionally, this energy is almost half of that calculated for 1,3-diaminopropan-2-ol, suggesting differing photophysical behaviors between these compounds. In the two compounds, 2-(nitromethylene)hexahydropyrimidin-5-ol and (2-(nitromethylene)oxazolidin-5-yl)methanamine, our computations for the S_0_–S_1_ transition result in weak oscillator strength and light absorption. This indicates that these transitions could be difficult to observe in spectroscopic experiments. On the whole, the Δ*E*(S_0_–T_1_) gap energy decreases from the def2-TZVP to the cc-pVTZ basis set, which follows a different trend compared to 2-(nitromethylene)hexahydropyrimidin-5-ol [def2-TZVP > VTZ > 6-311G(d,p) instead of 6-311G(d,p) > VTZ > def2-TZVP].

### Theoretical NMR Spectrum

The chemical shifts for 2-(nitromethylene)hexahydropyrimidin-5-ol that were determined experimentally in earlier sections were verified by DFT calculations. For this, two distinct basis sets and functionals were employed. A comparison between theoretical and experimental results is shown in Table 3, with the numbering of each atom visualized in Figure 6, compound **5**. In general, the numerical value of the ^13^C chemical shift is considerably influenced by the choice of functional and basis set, whereas the values of the ^1^H chemical shift are not significantly affected. As can be seen, we identified that the methods BP86/def2-QZVP and B3LYP/pcSseg-2 produce similar results. However, the values obtained from these methods slightly deviate from the experimental data. For example, the experimental chemical shift of the C_1_ atom, which is in the ring, presents the most intense peak at 44.57 ppm (see Figure S2B in the SM). The corresponding values calculated using BP86/def2-QZVP and B3LYP/pcSseg-2 are 53.43 and 52.73 ppm, respectively. These DFT calculations overestimate the experimental measurement by about 20%. The C_16_ atom is outside the ring and is bonded to the amino group. Our δ value calculated at B3LYP/pcSseg-2 for this atom differs by about 6.3 ppm from the experimental measurement (98.47 ppm). The lowest peak in the spectrum is attributed to the C_6_ atom, which has an experimental δ value of 154.23 ppm.

**Table 3:** Theoretically Calculated and Experimentally Determined Chemical Shifts (in ppm) for ^13^C and ^1^H NMR Spectra of 2-(Nitromethylene)hexahydropyrimidin-5-ol

atom number	theoretical methods					exp.
	B3LYP/def2-TZVP	BP86/def2-QZVP	B3LYP/pcSseg-2	BP86/def2-QZVP	B3LYP/def2-TZVP	
				(DMSO)	(DMSO)	
C_1_	48.49	53.43	52.73	54.48	50.23	44.57
C_2_	65.77	71.13	70.40	72.322	67.38	58.38
C_3_	44.83	49.12	48.58	51.05	47.52	44.57
C_6_	154.38	152.17	161.22	156.67	159.15	154.23
C_16_	101.77	103.36	104.80	107.76	106.36	98.47
H_9_	4.09	4.36	4.16	4.60	4.23	4.02
H_10_–H_13_	3.14	3.14	3.09	3.37	3.20	3.10–3.39
H_15_	9.82	10.50	10.48	10.66	9.17	8.83
H_17_	6.05	6.32	6.23	6.83	6.47	6.28

Examining Table 3, we also identify that the best theoretical results are provided by the B3LYP/def2-TZVP level of theory. The calculated ^13^C NMR chemical shifts are δ(C_1_) = 48.49 ppm, δ(C_2_) = 65.77 ppm, δ(C_3_) = 44.83 ppm, δ(C_6_) = 154.38 ppm, and δ(C_16_) = 101.77 ppm. These quantities differ by about 9, 13, 0.6, 0.1, and 3%, respectively, from the experimental measurements. Therefore, no improvement was observed when the size of the basis set was increased from def2-TZVP to def2-QZVP, demonstrating that the results can be adequately described by the selected base set. As can be seen, solvent effects on 2-(nitromethylene)hexahydropyrimidin-5-ol significantly influence the accuracy of the calculated ^13^C NMR chemical shifts. The differences between the B3LYP/def2-TZVP(DMSO) chemical shifts for carbons C_1_, C_2_, C_3_, C_6_, and C_16_ and experimental ones are 5.6, 9, 2.9, 4.9, and 7.9 ppm, respectively. The theoretical data determined using BP86/def2-QZVP in DMSO are somewhat less accurate.

For the calculated ^1^H NMR spectrum, the hydrogens H_10_-H_13_ are close and present a similar sign, see SM, with an experimental δ of 3.10–3.39 ppm in nice agreement with DFT prediction. The most intense peak in the spectrum is shown for hydrogen H_17_ (see Figure 2A in the SM). The experimental δ(H_17_) is 6.28 ppm, but the calculated value at B3LYP/def2-TZVP is 6.05 ppm. All other calculations consistently overestimate the experimental observation. In addition, the obtained results agree well with experimental data reported by Foks et al.^4^

In light of these positive outcomes, we have repeated DFT calculations to predict the ^1^H and ^13^C NMR chemical shifts of the other nonexperimentally observed compound (2-(nitromethylene)oxazolidin-5-yl)methanamine. All results are given in Table 4. The ^13^C NMR spectrum must have five peaks. Broadly speaking, the BP86/def2-QZVP shifts are 1.8–8.5 ppm larger than those obtained with the B3LYP/def2-TZVP level of theory, presumably the most accurate. All DFT computations highlight that δ(C_4_), in the aromatic region, presents the highest chemical shift while C_1_ has a lower δ value. Taking into account calculations without solvent effects, we found that the chemical shift of C_10_, in the vicinity of the nitro group, varies by less than 3 ppm. In comparison, the δ(C_10_) value in (2-(nitromethylene)oxazolidin-5-yl)methanamine is similar to the δ(C_16_) value in 2-(nitromethylene)hexahydropyrimidin-5-ol due to their specific geometric positions. We notice the same resemblance for δ(H_11_) in compound **6** and δ(H_17_) in compound **5**.

**Table 4:** Theoretically Calculated Chemical Shifts (in ppm) for ^13^C and ^1^H NMR Spectrum of (2-(Nitromethylene)oxazolidin-5-yl)methanamine

atom number	theoretical methods				
	B3LYP/def2-TZVP	BP86/def2-QZVP	B3LYP/pcSseg-2	BP86/def2-QZVP	B3LYP/def2-TZVP
				(DMSO)	(DMSO)
C_1_	46.03	52.37	51.83	52.08	45.83
C_2_	89.98	98.45	97.81	102.67	94.18
C_4_	166.05	167.86	174.67	170.08	169.50
C_10_	102.79	106.64	107.74	104.93	100.98
C_15_	48.88	54.86	54.83	54.26	48.44
H_6_	2.69	3.90	3.34	5.80	4.73
H_7_	2.32	3.20	2.70	3.54	2.75
H_8_	2.77	3.62	3.16	4.09	3.29
H_9_	3.94	4.78	4.29	5.33	4.53
H_11_	5.70	6.76	6.24	7.07	6.16

### DFT Investigation on the Reaction Mechanism of 2-(Nitromethylene)hexahydropyrimidin-5-ol Formation

**Figure 8:**
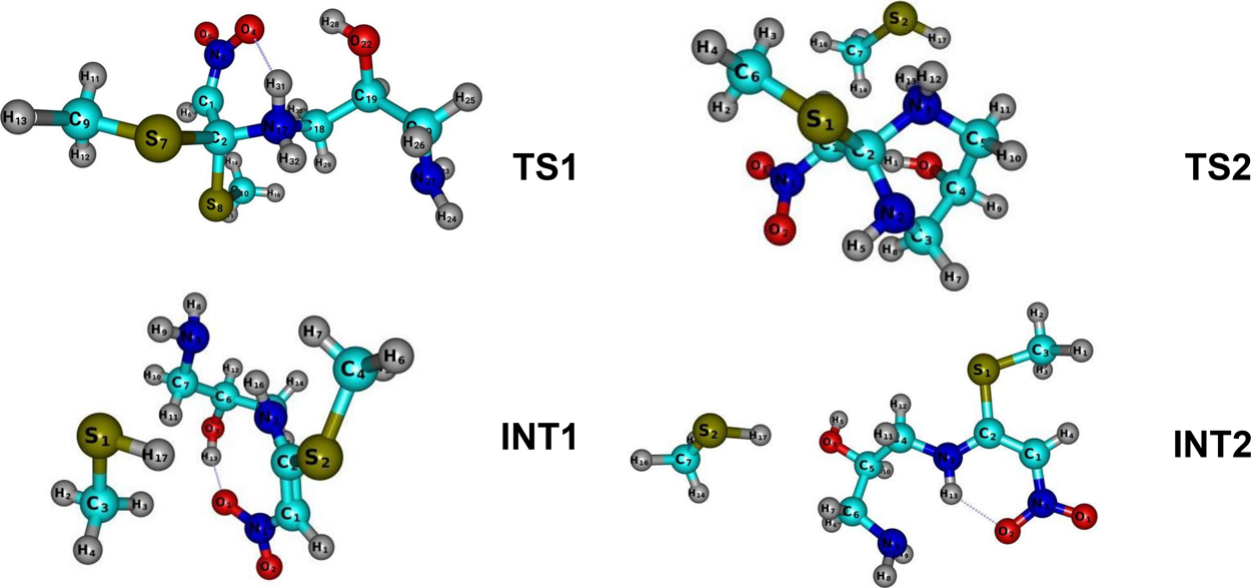
Optimized geometries of transition states, **TS1** and **TS2**, and intermediates, **INT1** and **INT2**, at the B3LYP/def2-TZVP level of theory.

From the perspective of DFT, we propose, for the first time, a mechanism for the reaction between 1,3-diaminopropan-2-ol and 1,1-bismethylsulfanyl-2-nitroethylene. Here, energetic, geometric, and frequency properties for the stationary states were computed at the B3LYP-def2-TZVP level of theory (see Tables S1–S9 of the SM). Vibrational harmonic frequencies and zero-point energies (ZPEs) for all stationary points calculated in this work are listed in Tables S10–S13 of the Supporting Information. Using theoretical calculations to unveil the details of this reaction, we identified at least two transition states in the formation of 2-(nitromethylene)hexahydropyrimidin-5-ol. The corresponding transition states were confirmed by the presence of only one imaginary frequency. In addition, two intermediates are also predicted. The geometry of these structures is depicted in Figure 8.

**Figure 9:**
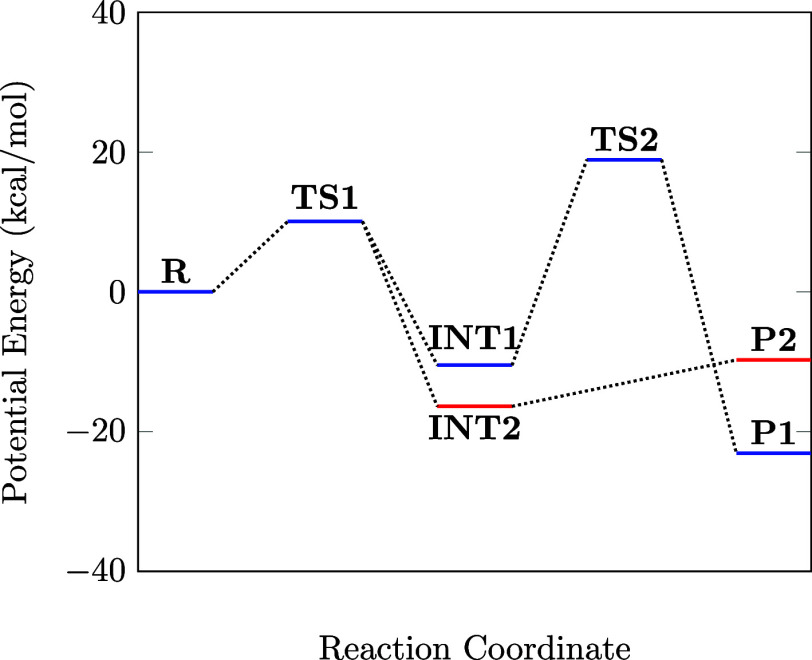
B3LYP/def2-TZVP energy profile for the formation of compounds **5** (blue) and **6** (red) with ZPE correction. Here, **R** represents the reactants (**4** + **1**), **P1** is the product 2-(nitromethylene)hexahydropyrimidin-5-ol plus two methanethiols, and **P2** refers to (2-(nitromethylene)oxazolidin-5-yl)methanamine plus two methanethiols. The final product structure **P1 (P2)** is shown in Figure 6 as represented by **5** (**6**).

Our frequency analysis reveals that the transition state, denoted here as **TS1**, presents one imaginary frequency of −210 cm^–1^. We also find one imaginary frequency for a second transition state, **TS2**, of −48 cm^–1^. As can be seen, **TS2** presents a van der Waals structure, which justifies the low imaginary frequency value. Consequently, its contribution to the ZPE computation is negligible. The energy profile of the reaction mechanism, relative to the entrance of 1,3-diaminopropan-2-ol and 1,1-bismethylsulfanyl-2-nitroethylene, is presented in Figure 9, with ZPE corrections, where two reaction channels are displayed on it. The first pathway, shown in blue, corresponds to the formation of 2-(nitromethylene)hexahydropyrimidin-5-ol, while the second pathway, shown in red, leads to the formation of (2-(nitromethylene)oxazolidin-5-yl)methanamine.

The initial step in both pathways is the formation of **TS1**, which is higher in energy than the separated reactants by Δ*E* + ΔZPE = 10.0 kcal/mol. The ZPE energies calculated at B3LYP/def2-TZVP are 89.6 (**4**), 70.6 (**1**), and 161.5 kcal/mol (**TS1**). The transition state **TS1** connects the **4** + **1** entrance to the intermediates denoted here as **INT1** and **INT2** in an exothermic process. Therefore, the energetics of **TS1** appear to be very important to deciphering the preferred mechanism. In terms of the frontier molecular orbitals, our computations show that the intermediate **INT1** has a HOMO–LUMO energy gap of 4.19 eV while the intermediate **INT2** has an energy gap of 4.57 eV. Note also that **INT1** is energetically Δ*E* + ΔZPE = 10.50 kcal/mol below the energy of the reactants, while **INT2** lies 16.39 kcal/mol below the energy of the reactants. The formation of intermediate **INT1** releases less energy than **INT2**, which could explain why our experiment observes the formation of 2-(nitromethylene)hexahydropyrimidin-5-ol instead of (2-(nitromethylene)oxazolidin-5-yl)methanamine.

In terms of molecular geometry, the pathway **TS1** → **INT1**/**INT2** is characterized by the formation of one methanethiol (−CH_3_SH). The major structural difference between them is that **INT1** presents an open six-membered heterocycle, while **INT2** features an open five-membered heterocycle. To form **INT1**, the S_8_–C_2_ bond in **TS1** must first be broken. During this process, the hydrogen H_31_ bonded to nitrogen N_17_ is attracted to S_8_ through electrostatic interactions, thus producing CH_3_SH as shown in the scheme in Figure 4.

Following the blue pathway, the calculations indicate that **INT1** → **TS2** corresponds to an endothermic process. In this step, the nitrogen belonging to the amine group in **INT1** attacks the carbon C_2_ to form a six-membered heterocycle characteristic in the **TS2** structure. Energetically, **TS2** lies 18.90 kcal/mol above the reactants with a barrier energy of 29.40 kcal/mol. The product **P1** corresponds to the 2-(nitromethylene)hexahydropyrimidin-5-ol plus two methanethiols formation, where the **TS2** → **P1** passage is exothermic, releasing 42 kcal/mol. In this process, the S_1_–C_2_ bond in **TS2** is broken, and the NH_2_ group releases one hydrogen, yielding a new molecule of methanethiol.

For the formation of (2-(nitromethylene)oxazolidin-5-yl)methanamine, the calculated reaction path, shown in red, differs somewhat from that in blue. The present DFT calculations did not find a transition state between **INT2** and **P2**. However, from the reaction profile (Figure 9), one can observe that the pathway in red must be exothermic (−9.75 kcal/mol). Our results corroborate the experiment, which just saw the chemical 2-(nitromethylene)hexahydropyrimidin-5-ol as a product. Based on the benchmark baseline, B3LYP/def2-TZVP is the converged one deemed to be in excellent agreement with the RMN results.

## Conclusions

Because of its broad application, understanding the chemical interactions of ketene dithioacetals is important in this research field. In this work, 2-(nitromethylene)hexahydropyrimidin-5-ol was studied both experimentally and theoretically by DFT calculations. From the experimental side, this compound was produced by the reaction between 1,3-diaminopropan-2-ol and 1,1-bismethylsulfanyl-2-nitroethylene and promptly identified by an NMR experiment. The experimental chemical shifts obtained for 2-(nitromethylene)hexahydropyrimidin-5-ol were compared with theoretical values calculated both with and without solvent effects, allowing us to examine the influence of the environment on the NMR parameters. However, to our surprise, the best results were achieved when no solvent effects were considered, using a B3LYP/def2-TZVP level of theory (Table 3). Consequently, the same functional and basis sets were used to predict the ^13^C and ^1^H NMR chemical shifts for (2-(nitromethylene)oxazolidin-5-yl)methanamine, as shown in Table 4. To the best of our knowledge, this is the first work to report NMR data for this species.

We were experimentally unable to find any evidence of 2-(nitromethylene)oxazolidin-5-yl)methanamine in our investigations. Therefore, a reaction mechanism based on DFT calculations at the B3LYP/def2-TZVP level of theory was proposed to explain why this occurs. Our calculations predict the formation of a transition state, **TS1**, after the reaction of reactants. This channel yields the **INT1**/**INT2** product, thereby overcoming an energy barrier of 10.0 kcal/mol. Two different pathways emerge in this step. The first pathway, originating from the formation of **INT1**, results in one 2-(nitromethylene)hexahydropyrimidin-5-ol and two methanethiols, identified as product **P1** in Figure 9 (blue). This reaction is exothermic, with the energy of the products being −23.10 kcal/mol below that of the reactants. However, it is shown in such a Figure that the **TS2** transition state connects the intermediate **INT1** and product **P1**, overcoming an energy barrier of about 42 kcal/mol relative to the **INT1** structure. From an energetic point of view, it is evident that intermediate **INT2** appears to be more stable than **INT1**, which aligns with its corresponding gap energy values. However, the formation of **INT1** requires less energy release compared to **INT2**, indicating a more favorable reaction mechanism. Although we searched extensively, we did not find a transition state connecting **INT2** and the product **P2**, which corresponds to the formation of one (2-(nitromethylene)oxazolidin-5-yl)methanamine and two metanethiols. The formation of **P2** is also exothermic, with the energy of the products estimated to be −9.75 kcal/mol below that of the reactants.

The theoretical results appear to correlate closely with the NMR experimental data, and the proposed reaction mechanism is reasonably well described by the B3LYP functional. Through theory and experimentation, our work clarifies the reaction pathway of ketene dithioacetals, advancing our understanding of this chemical process.

## Supporting Information Available

Total energies, optimal geometries, vibrational frequencies, dipole moments, NMR spectra, and IR spectra produced in this work (PDF)
