# Supplementary material for: Understanding 2-(Nitromethylene)hexahydropyrimidin-5-ol Reaction Processes and NMR Spectroscopy: A Theoretical and Experimental Investigation
Source: ACS Omega. 2024 Dec 21;10(1):995–1005. doi: 10.1021/acsomega.4c08242 (PMC11740371; doi:10.1021/acsomega.4c08242)
Supplement: Supplementary file 1 — ao4c08242_si_001.pdf [file ao4c08242_si_001.pdf]

Understanding 2-(nitromethylene)hexahydropyrimidin-5-ol reaction processes and NMR spectroscopy: A theoretical and experimental investigation
Supplementary Material

Ramon S. da Silva[1],* Diego P. Sangi[2], Rodrigo G. Amorim[1]

[1]*Departamento de Física, Instituto de Ciências Exatas - ICEx, Universidade Federal Fluminense, Volta Redonda, Rio de Janeiro, Brazil*

[2]*Departamento de Química - Instituto de Ciências Exatas - ICEx, Universidade Federal Fluminense, Volta Redonda, Rio de Janeiro, Brazil*

* ramonsousa@id.uff.br

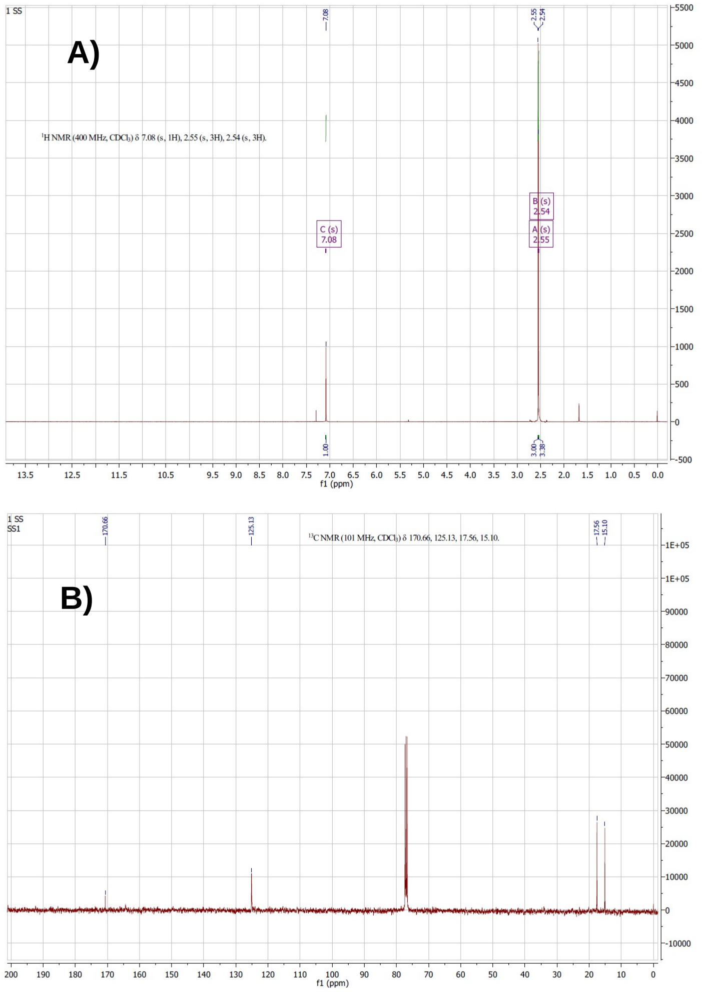

Figure S1. The experimental A) ^{1}H and B) ^{13}C NMR spectrum of 1,1-bis-methylsulfanyl-2-nitroethylene.

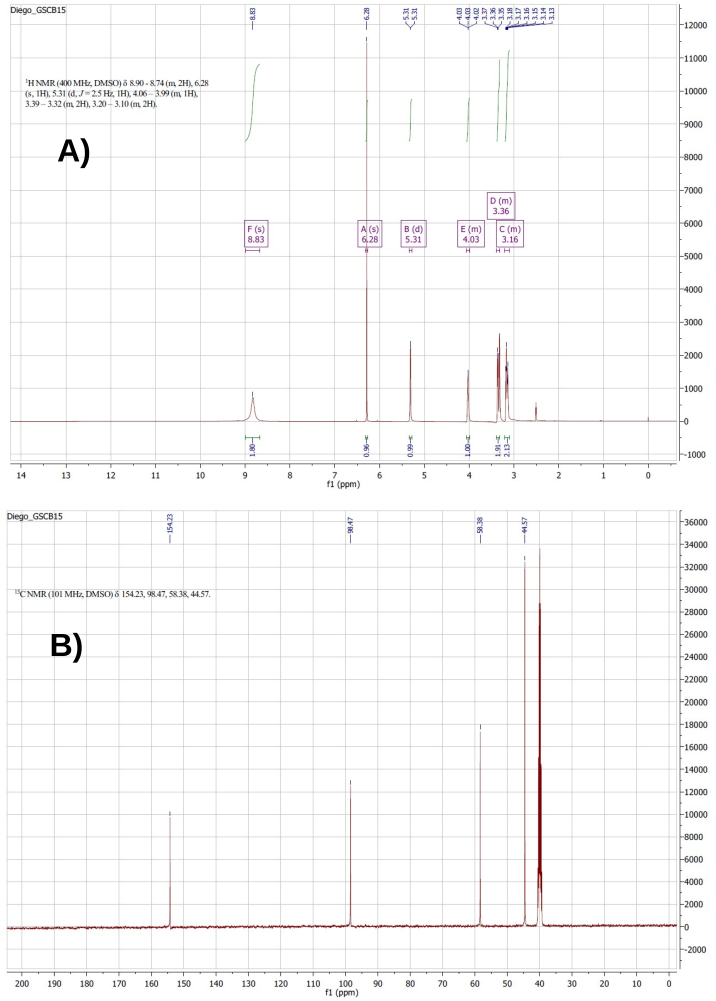

Figure S2. The experimental A) ^{1}H and B) ^{13}C NMR spectrum of 2-(nitromethylene)hexahydropyrimidin-5-ol.

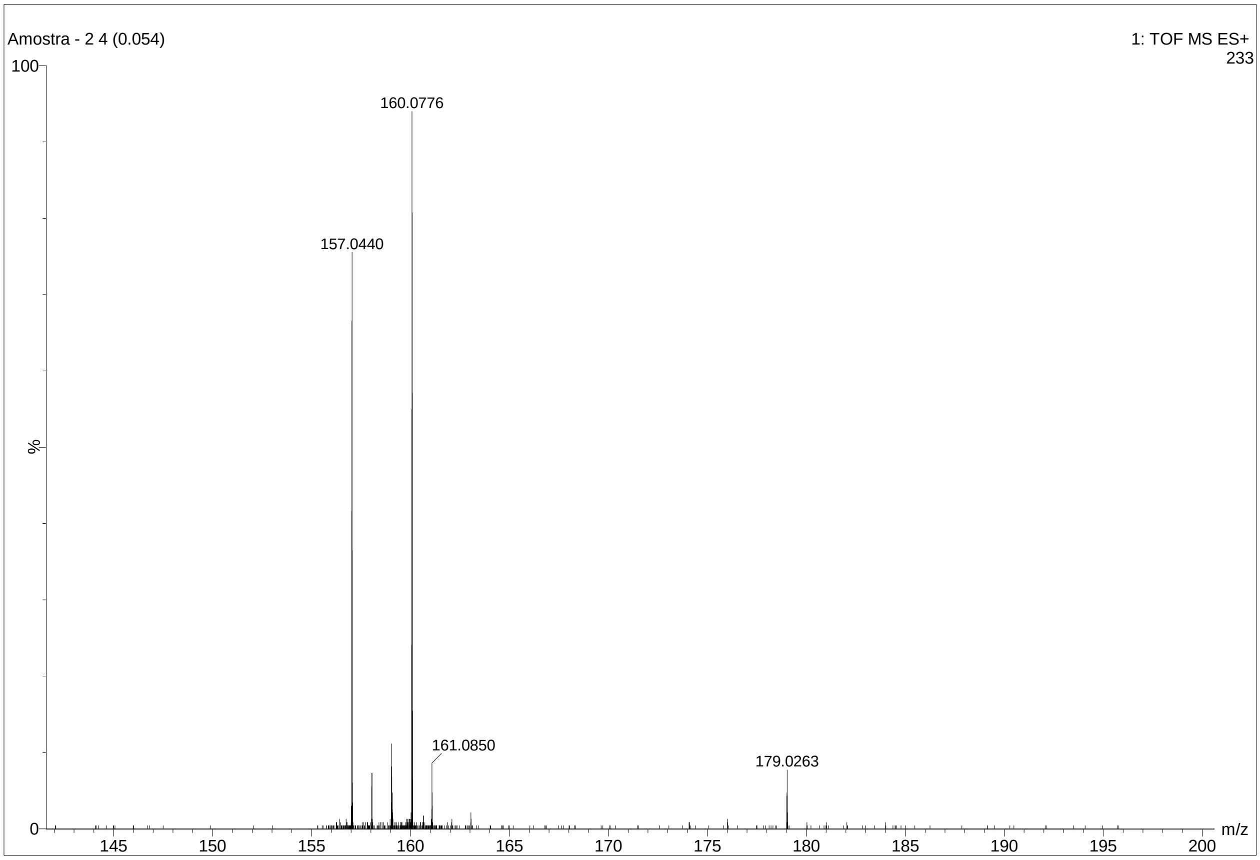

Figure S3. High-resolution mass spectrometry (HRMS) analysis of 2-(nitromethylene)hexahydropyrimidin-5-ol using a Shimadzu GCMS-QP2010 Plus and a Waters/Micromass UPLC-QTof-MS instrument.

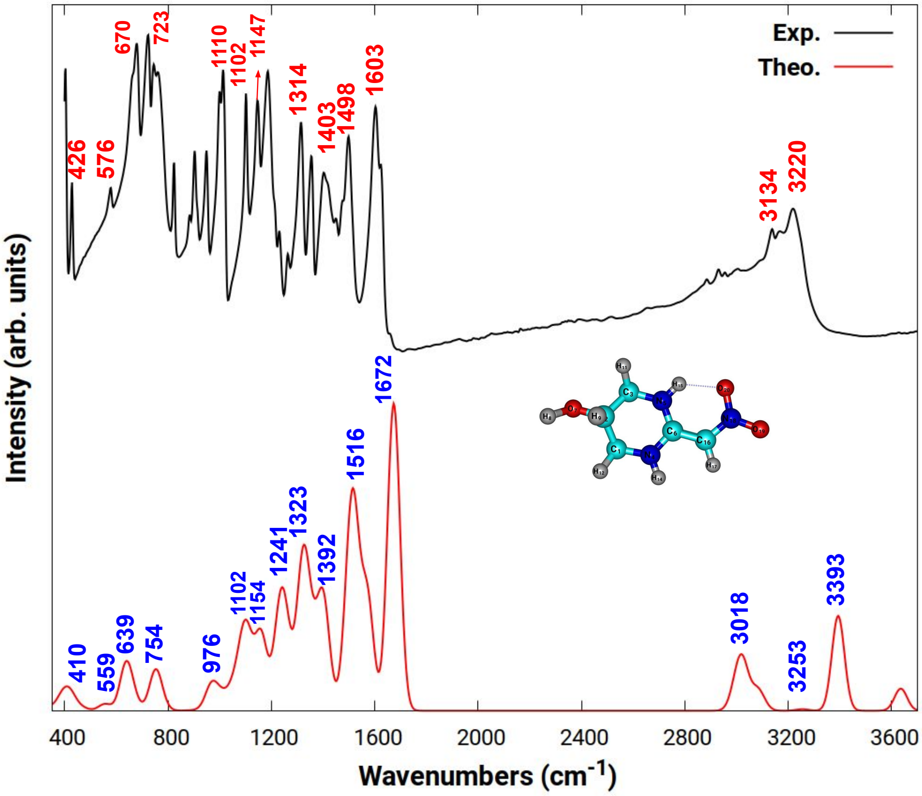

Figure S4. A comparison between the experimental and calculated (B3LYP/def2-TZVP) IR spectra of 2-(nitromethylene)hexahydropyrimidin-5-ol.

Table S1. Comparison of the calculated bond lengths (in Å), bond angles (in degree), total energies (in Hartree), and dipole moments (in Debye) of 1,3-diaminopropan-2-ol, compound **4** calculated using the B3LYP functional.

Bond lengths	6-311G(d,p)	def2-TZVP	VTZ
C_1-C_2	1.535	1.532	1.530
C_2-C_3	1.535	1.532	1.530
C_1-N_4	1.462	1.459	1.460
C_2-O_6	1.478	1.443	1.441
C_1-H_{13}	1.093	1.094	1.093
N_5-H_9	1.011	1.014	1.014
O_6-H_{16}	0.969	0.960	0.958
Bond angles			
C_1-C_2-C_3	113.5	112.2	112.4
C_2-C_3-N_5	115.5	116.1	116.0
C_2-O_6-H_{16}	111.0	109.5	109.4
H_7-N_4-H_8	112.2	107.3	106.9
H_{14}-C_3-H_{15}	106.9	106.7	106.7
Energy			
	-304.848133	-304.984774	-304.989487
dipole moment			
	3.312	2.982	2.788

Table S2. Comparison of the calculated bond lengths (in Å), bond angles (in degree), total energies (in Hartree), and dipole moments (in Debye) of 1,1-bismethylsulfanyl-2-nitroethylene, compound **1** calculated using the B3LYP functional.

Bond lengths	6-311G(d,p)	def2-TZVP	VTZ
C_1-C_2	1.356	1.354	1.354
C_1-N_3	1.442	1.440	1.436
C_2-S_8	1.781	1.783	1.773
C_2-S_7	1.753	1.756	1.745
C_1-H_6	1.079	1.078	1.076
N_3-O_5	1.230	1.232	1.229
C_{10}-H_{14}	1.089	1.088	1.086
Bond angles			
N_3-C_1-C_2	127.2	127.2	127.2
O_5-N_3-O_4	124.3	124.0	124.1
C_2-S_7-C_9	106.0	105.9	106.5
C_1-C_2-S_8	114.1	113.8	113.7
Energy			
	-1157.919483	-1157.944435	-1157.978348
dipole moment			
	5.258	5.594	5.347

Table S3. Comparison of the calculated bond lengths (in Å), bond angles (in degree), total energies (in Hartree), and dipole moments (in Debye) of 2-(nitromethylene)hexahydropyrimidin-5-ol, compound **5** calculated using the B3LYP functional

Bond lengths	6-311G(d,p)	def2-TZVP	VTZ
N_{18}-O_{19}	1.234	1.237	1.233
C_{16}-H_{17}	1.077	1.076	1.074
C_6-C_{16}	1.394	1.393	1.391
C_6-N_4	1.369	1.367	1.366
O_7-H_8	0.962	0.963	0.961
Bond angles			
C_6-C_{16}-N_{18}	124.0	124.1	124.1
O_{20}-N_{18}-O_{19}	122.0	121.7	121.8
C_6-N_4-H_{14}	116.0	116.3	116.1
C_2-O_7-H_8	108.8	109.3	109.4
Energy			
	-585.619566	-585.671971	-585.682264
dipole moment			
	8.752	9.116	8.730

Table S4. Comparison of the calculated bond lengths (in Å), bond angles (in degree), total energies (in Hartree), and dipole moments (in Debye) of 2-(nitromethylene)oxazolidin-5-yl)methanamine, compound **6** calculated using the B3LYP functional

Bond lengths	6-311G(d,p)	def2-TZVP	VTZ
N_{12}-O_{14}	1.235	1.238	1.234
C_{10}-N_{12}	1.421	1.418	1.416
C_4-C_{10}	1.361	1.360	1.359
N_5-H_6	1.008	1.007	1.006
C_1-H_7	1.098	1.097	1.096
Bond angles			
C_{10}-C_4-N_5	124.1	124.0	124.0
O_{14}-N_{12}-O_{13}	124.1	123.7	123.9
C_4-N_5-H_6	118.8	119.0	118.6
H_7-C_1-H_8	109.1	109.2	108.9
Energy			
	-585.598355	-585.650306	-585.661993
dipole moment			
	8.591	8.997	8.592

Table S5. Comparison of the calculated bond lengths (in Å), bond angles (in degree), total energies (in Hartree), and dipole moments (in Debye) of methanethiol calculated using the B3LYP functional.

Bond lengths	6-311G(d,p)	def2-TZVP	VTZ
C_1-S_2	1.836	1.840	1.830
C_1-H_3	1.089	1.088	1.087
S_2-H_6	1.348	1.350	1.345
Bond angles			
H_4-C_1-H_5	108.8	108.8	108.8
C_1-S_2-H_6	96.9	96.8	97.1
Energy			
	-438.645300	-438.644504	-438.658418
dipole moment			
	1.7144	1.667	1.547

Table S6. Comparison of the calculated bond lengths (in Å), bond angles (in degree), total energies (in Hartree), and dipole moments (in Debye) of transition state (TS1), structure **7** calculated using the B3LYP functional.

Bond lengths	6-311G(d,p)	def2-TZVP	VTZ
S_8-C_{10}	1.837	1.842	1.830
C_1-C_2	1.463	1.464	1.486
C_{10}-H_{15}	1.091	1.090	1.089
N_3-O_5	1.235	1.239	1.226
N_{21}-H_{24}	1.014	1.012	1.011
Bond angles			
H_{24}-N_{21}-H_{23}	106.6	107.0	106.6
C_2-C_1-N_3	123.4	123.5	125.8
O_4-N_3-O_5	119.4	118.9	114.2
C_2-N_{17}-H_{32}	106.7	109.9	109.1
C_{19}-O_{22}-H_{28}	110.9	111.4	110.2
C_2-S_8-C_{10}	105.9	105.9	106.3
Energy			
	-1462.866554	-1462.915142	-1462.960890
dipole moment			
	6.868	7.234	4.923

Table S7. Calculated bond lengths (in Å), bond angles (in degree), total energies (in Hartree), and dipole moments (in Debye) for INT1 at B3LYP/def2-TZVP level of theory

Bond lengths	
S_2-C_2	1.837
S_1-C_3	1.843
C_4-H_6	1.091
C_1-C_2	1.378
N_1-O_2	1.233
N_3-H_9	1.014
O_3-H_{13}	0.968
Bond angles	
H_8-N_3-H_9	106.6
C_2-C_1-N_3	123.4
O_4-N_3-O_5	119.4
C_2-N_2-H_{16}	106.7
C_6-O_3-H_{13}	110.9
C_2-S_2-C_4	105.9
Energy	
	-1462.946978
dipole moment	
	7.094

Table S8. Calculated bond lengths (in Å), bond angles (in degree), total energies (in Hartree), and dipole moments (in Debye) for INT2 at B3LYP/def2-TZVP level of theory

Bond lengths	
S_3-C_2	1.792
C_1-C_2	1.379
C_1-H_4	1.072
C_1-C_2	1.378
N_2-H_{13}	1.023
N_1-O_1	1.251
O_3-H_5	0.963
Bond angles	
O_1-N_1-O_2	122.6
H_8-N_3-H_9	107.7
C_7-S_2-H_{17}	97.0
C_2-N_2-C_4	127.3
C_5-O_3-H_5	109.4
Energy	
	-1462.955040
dipole moment	
	5.267

Table S9. Calculated bond lengths (in Å), bond angles (in degree), total energies (in Hartree), and dipole moments (in Debye) for TS2 at B3LYP/def2-TZVP level of theory

Bond lengths	
S_1-C_6	1.830
C_1-C_2	1.473
N_1-O_2	1.247
S_2-H_{17}	1.350
N_3-H_{12}	1.022
O_3-H_1	0.984
Bond angles	
O_1-N_1-O_2	121.9
C_2-N_2-C_3	114.0
C_2-S_1-C_6	102.3
Energy	
	-1462.902202
dipole moment	
	8.708

Table S10. Harmonic vibrational frequencies (in cm^{-1}) and zero point energies (in kcal/mol) calculated at B3LYP/def2-TZVP.

Species						
**1,3-diaminopropan-2-ol**						
	191.59	291.97	301.80	330.73	400.23	462.89
	545.17	799.13	825.15	860.45	867.07	984.27
	998.64	1069.18	1070.26	1120.93	1158.82	1172.10
	1288.61	1292.11	1384.84	1393.37	1394.51	1399.53
	1417.70	1473.41	1483.86	1664.30	1665.24	2974.88
	3023.92	3033.61	3061.83	3073.83	3484.93	3485.44
	3569.20	3569.59	3860.21			
ZPE	89.62					
**1,1-bismethylsulfanyl-2-nitroethylene**						
	47.67	59.27	103.96	123.51	149.18	167.43
	185.82	222.92	267.25	273.21	353.92	417.63
	523.99	678.08	685.35	711.01	724.05	747.79
	812.88	892.91	971.96	975.80	985.66	998.75
	1012.15	1308.06	1323.83	1358.53	1360.60	1456.40
	1465.42	1480.35	1485.07	1516.85	1580.73	3057.46
	3060.21	3142.76	3143.72	3166.08	3170.82	3241.66
ZPE	70.64					
**2-(Nitromethylene)hexahydropyrimidin-5-ol**						
	55.13	90.85	141.88	156.95	172.35	226.22
	234.26	320.83	355.81	389.34	420.00	465.69
	545.35	557.39	638.05	669.79	742.45	748.78
	753.37	784.49	873.20	921.66	968.95	1009.50
	1055.16	1093.81	1108.85	1139.87	1159.85	1222.27
	1231.74	1256.22	1311.60	1315.14	1325.74	1359.62
	1390.56	1402.77	1452.49	1491.11	1504.62	1516.22
	1525.94	1571.01	1673.27	2996.29	3008.12	3023.86
	3075.29	3094.38	3254.85	3393.29	3635.65	3816.24
ZPE	99.57					

Table S11. Harmonic vibrational frequencies (in cm^{-1}) and zero point energies (in kcal/mol) calculated at B3LYP/def2-TZVP.

Species						
**2-(nitromethylene)oxazolidin-5-yl)methanamine**						
	48.63	77.78	96.32	110.91	157.42	193.73
	227.49	305.02	354.20	393.35	430.24	471.46
	485.08	664.26	673.56	705.65	755.89	760.02
	786.02	830.70	861.47	908.28	988.45	1021.77
	1029.43	1065.92	1075.15	1099.03	1126.94	1197.05
	1235.85	1240.92	1279.80	1332.34	1352.20	1366.35
	1397.86	1413.68	1440.10	1451.24	1476.12	1519.57
	1530.60	1673.85	1679.12	2997.24	3026.04	3035.12
	3069.26	3100.52	3256.56	3497.34	3578.62	3625.45
ZPE	99.32					
**Methanethiol**						
	236.41	690.71	797.41	973.10	1094.59	1368.25
	1477.27	1490.09	2653.58	3055.01	3137.76	3142.24
ZPE	28.76					
**Transition State 1**						
	210.17i	36.11	51.12	61.93	70.42	80.99
	88.57	106.57	131.52	146.29	153.07	160.94
	168.22	190.89	209.84	232.54	258.33	282.82
	325.75	334.77	368.19	381.59	407.02	467.25
	490.52	527.96	615.95	662.54	674.83	682.74
	704.97	720.84	751.31	761.61	811.68	866.45
	869.14	892.23	903.78	962.55	971.55	978.95
	981.39	984.67	995.88	1038.08	1066.20	1101.38
	1103.80	1133.37	1164.78	1192.11	1274.71	1300.58
	1317.86	1349.02	1352.21	1354.21	1361.72	1377.87
	1406.18	1415.05	1441.45	1452.90	1464.99	1465.53
	1486.67	1487.82	1505.64	1512.84	1519.38	1595.79
	1667.72	2058.58	2955.30	3005.80	3051.79	3054.44
	3055.43	3082.93	3136.58	3139.81	3140.56	3147.37
	3151.55	3270.04	3488.98	3502.02	3582.30	3688.42
ZPE	161.47					

Table S12. Harmonic vibrational frequencies (in cm^{-1}) and zero point energies (in kcal/mol) calculated at B3LYP/def2-TZVP.

Species						
**Intermediate 1**						
	17.21	28.64	31.70	47.26	52.54	59.08
	64.91	68.43	81.83	109.50	130.40	146.62
	159.16	168.04	182.72	201.03	245.26	280.26
	284.25	295.14	327.22	352.92	373.22	448.52
	479.39	501.84	564.86	579.07	628.61	684.02
	685.33	707.27	725.57	757.48	773.21	799.64
	804.71	858.53	903.24	947.32	970.85	978.54
	981.16	986.18	1009.50	1030.92	1045.19	1090.20
	1098.15	1115.00	1179.71	1193.21	1256.47	1299.09
	1312.90	1339.60	1353.29	1366.77	1372.68	1374.28
	1394.93	1424.26	1454.54	1467.72	1477.12	1479.80
	1487.40	1496.07	1505.60	1512.86	1551.53	1612.47
	1649.75	2642.15	3016.03	3020.81	3037.23	3055.77
	3057.39	3104.95	3140.94	3145.47	3146.60	3150.05
	3152.44	3265.42	3447.16	3481.96	3558.56	3701.18
ZPE	160.89					
**Transition State 2**						
	-47.90	28.86	36.98	51.24	73.31	77.19
	85.61	103.20	113.08	120.11	131.29	143.77
	167.46	211.09	254.78	277.18	307.79	329.08
	346.17	379.99	391.87	404.38	425.82	503.12
	575.04	605.72	648.06	677.71	697.36	704.37
	724.77	761.15	780.92	796.81	797.53	804.83
	851.40	891.22	943.35	970.53	974.99	979.87
	982.05	1003.07	1020.50	1028.25	1083.26	1091.19
	1112.13	1133.60	1190.23	1247.94	1259.83	1274.21
	1322.50	1336.82	1347.67	1364.01	1369.56	1377.47
	1381.44	1404.82	1435.89	1460.34	1470.61	1474.74
	1476.84	1482.36	1486.14	1489.74	1502.06	1515.62
	1643.16	2656.35	3027.43	3053.25	3064.31	3069.96
	3077.41	3087.12	3131.87	3143.94	3153.68	3158.31
	3167.72	3200.51	3216.45	3380.49	3419.73	3541.04
ZPE	162.21					

Table S13. Harmonic vibrational frequencies (in cm^{-1}) calculated at B3LYP/def2-TZVP for the reactants, transition states, and products.

Species						
**Intermediate 2**						
	5.36	10.83	17.46	24.00	31.78	36.49
	56.97	72.32	79.12	95.51	108.93	136.29
	147.84	167.37	179.84	230.80	238.07	254.78
	268.82	280.95	331.37	334.66	370.16	378.22
	419.25	472.93	536.70	551.54	581.81	666.50
	693.22	709.15	730.48	743.75	807.13	820.50
	828.01	848.34	874.95	911.35	971.24	975.55
	987.34	996.58	1023.31	1053.73	1074.64	1104.62
	1108.52	1128.10	1157.96	1202.89	1250.78	1292.51
	1306.57	1329.90	1349.58	1364.67	1366.58	1381.32
	1404.27	1415.60	1431.02	1450.24	1470.42	1477.66
	1489.96	1490.20	1494.50	1512.51	1530.28	1605.99
	1670.22	2630.26	2990.46	2997.73	3016.16	3054.20
	3055.98	3071.26	3080.29	3135.15	3140.90	3143.53
	3150.20	3305.53	3352.97	3515.75	3598.17	3807.84
ZPE	160.07					
